# Six hours positive pressure ventilation with size 5 laryngeal mask in a 55-kg patient

**DOI:** 10.4103/0019-5049.63631

**Published:** 2010

**Authors:** Jayashree Patki, C Naresh K Reddy

**Affiliations:** Department of Anaesthesiology, Krishna Institute of Medical Sciences, Secunderabad 500 003, India

Sir,

I read the letter written by Dr. Jayashree Sood in relation to an article by Dr. Kerem (6 hrs. Positive pressure ventilation with size 5 LMA). I would like to share my experience of using LMA (laryngeal mask airway) for a prolonged period, but in a spontaneously breathing patient. One ASA grade1 (wt. 60 kg) patient was posted for cosmetic surgery. General anaesthesia was planned and size 4 LMA was inserted under the effect of inj. propofol (120 mg), inj. fentanyl (150 *μ*g) and inj. midazolam (2 mg) thinking that it is a small procedure, because of some mis-communication between the surgeon and the anaesthetist. Then, we realized that it would take a much longer time. But still we decided to continue the procedure, instead of opting for intubation and controlled ventilation. The entire procedure lasted for 15 h. During this time, anaesthesia was maintained with O_2_:N_2_O (1 + 1l), sevoflurane (1–1.5%), propofol infusion 6 ml/h (1mg/kg/h), inj. fentanyl 25 *μ*g/h (0.5–1 *μ*g/kg/h), inj. midazolam (1 mg/2h) on Datex Ohmeda Aespire anaesthesia station with circle absorber. Last dose of midazolam and fentanyl was given 1 h before the end of the surgery. Propofol infusion was stopped 30 min before, sevoflurane 20 min before and N_2_ O 10 min before removing the LMA. We noticed that patient was very much awake, alert and oriented without any intraoperative awareness while shifting out of OT. There were no signs of delayed recovery.

The patient was discharged on the next day. There were no neurovascular or airway complications[1,2] as described in the literature. LMA cuff was intact after 15 h of continuous use. Propofol and midazolam are known to alter the levels of lipids, especially triglycerides and cholesterol. In the propofol group, Ilhan et al. observed a significant increase in triglycerides and very low-density lipoprotein levels 4 h postoperatively. In the midazolam group, they observed a significant decrease in low-density lipoprotein, cholesterol at the end of and 4 and 24 h postoperatively.[3] Sevoflurane is known for causing hepatic dysfunction and its nephrotoxicity. So, serum creatinine, liver function tests and lipid profile were repeated on first and third postoperative days. They were found to be within normal limits, as demonstrated by Myles[4] that a propofol infusion technique does not result in elevation of serum lipids and supports its increased popularity in the maintenance of anaesthesia.
